# Utilization of adjuvant arthritis model for evaluation of new approaches in rheumatoid arthritis therapy focused on regulation of immune processes and oxidative stress

**DOI:** 10.2478/v10102-011-0007-9

**Published:** 2011-03

**Authors:** Katarína Bauerová, Silvester Poništ, Danica Mihalová, František Dráfi, Viera Kuncírová

**Affiliations:** Institute of Experimental Pharmacology & Toxicology, Slovak Academy of Sciences, SK-84104 Bratislava, Slovak Republic

**Keywords:** arthritis, methotrexate, combination therapy, oxidative stress, carnosine, coenzyme Q10, pyridoindoles, stilbenoids

## Abstract

As a number of disease-modifying anti-rheumatic drugs often have side effects at high doses and/or during long-term administration, increased efficacy without increased toxicity is expected for combination therapy of rheumatoid arthritis (RA). The safety of long-term therapy of RA is very important as patients with RA are usually treated for two or more decades. This experimental overview is focused on some promising substances and their combinations with the standard antirheumatic drug – methotrexate (Mtx) for treatment of rheumatoid arthritis. The adjuvant arthritis model in Lewis rats was used for evaluation of antiinflammatory efficacy of the substances evaluated. Mtx was administered in the oral dose of 0.3 mg/kg b.w. twice a week. Natural and synthetic antioxidants were administered in the daily oral dose of 20 mg/kg b.w for coenzyme Q_10_ (CoQ_10_), 150 mg/kg b.w for carnosine (Carn), 15 mg/kg b.w. for stobadine dipalmitate (Stb) and its derivative SMe1.2HCl (SMe1), and 30 mg/kg b.w. for pinosylvin (Pin) or pterostilbene (Pte). Mtx in the oral dose of 0.4 mg/kg b.w. twice a week was combined with Pin in the oral daily dose of 50 mg/kg b.w. Clinical (hind paw volume – HPV), biochemical (activity of GGT in joint and level of TBARS in plasma), and immunological (IL-1 in plasma) parameters were assessed. Our results achieved with different antioxidants in monotherapies showed a reduction of oxidative stress in adjuvant arthritis independently of the chemical structure of the compounds. Pin was the most effective antioxidant tested in decreasing HPV. All combinations tested showed a higher efficacy in affecting biochemical or immunological parameters than Mtx administered in monotherapy. The findings showed the benefit of antioxidant compounds for their use in combination therapy with methotrexate.

## Introduction

This experimental overview is focused on some promising substances and their combinations with the standard antirheumatic drug methotrexate (Mtx) for treatment of rheumatoid arthritis (RA). Preclinical research on animal models of RA is very important for alerting the healthcare and scientific community, as well as pharmaceutical companies, of the existence of new or “forgotten” molecules. Most antirheumatics have side-effects when used in higher doses and/or within long-term dosage. Combinatory therapy is expected to have a higher efficacy without increased toxicity. Mtx has become the main immunosuppressive substance used in the treatment of patients with RA (Weinblatt, 2003). The use of Mtx has to be limited due to its toxic manifestations, *e.g.* abdominal disorder, alopecia, oral ulcers, and cytopenia (Alarcon *et al*., 1989). Ineffectiveness of treatment can be also observed. In the survey of McKendry and Dale (1993), due to the risk of treatment termination was substantiated in 75% of patients with RA taking Mtx for 60 months. An adverse drug effect proved to be a more common reason for treatment termination (53%) compared to loss/lack of beneficial effect (22%), other reasons (16%), or lost to follow-up (9%). On the other hand, the therapeutic efficacy of Mtx can be increased by combination with other synthetic drugs or inhibitors of TNF-α (Smolen *et al*., 2010). Application of biological therapy (antibodies or soluble receptors of TNF-α, IL-1 and IL-6) represents a great progress in the therapy of RA, while biological treatment is also frequently combined with Mtx (Maini *et al*., 1998; Weinblatt *et al*., 1999). There are countless possibilities for combinations with Mtx. Many substances were neglected when they failed to show good efficacy in monotherapy compared to standard antirheumatics. They would not get a second chance if the expected reduction of clinical parameters (mainly edema of joints) did not materialize, despite the fact that they improved many biochemical disease markers. This overview is focused mostly on substances of natural origin possessing antiinflammatory and antioxidative properties along with minimal side effects when administered to animals. The safety of long-term therapy of RA is very important as patients with RA are usually treated for two or more decades.

In our experiments of the last five years, synthetic (pyridoindoles) and natural substances (polysaccharides: glucomannan, beta-glucan; endogenous molecules: coenzyme Q_10_ and carnosine; different substances and products of plant origin) with immunomodulatory and antioxidative effects were assessed in monotherapy by using experimental adjuvant arthritis (AA). These active substances decreased the progression of AA when administered to arthritic rats over the period of 28 days (Bauerova *et al*., 2005a; 2005b; 2008a; 2008b; 2009; Drabikova *et al*., 2009; Drafi *et al*., 2010; Jančinova *et al*., 2009; Kogan *et al*., 2005; Mačičkova *et al*., 2010; Poništ *et al*., 2010; Sotnikova *et al*., 2009; Štrosova *et al*., 2008, 2009). Recently we focused on combined therapy with Mtx and confirmed the potent effect of this drug in several experiments administered along with an appropriate antioxidative substance (Bauerova *et al*., 2010; Jančinova *et al*., 2010; Rovensky *et al*., 2009a).

The primary beneficial effect concerned the reduction of articular edema and normalization of biochemical parameters were also reported by other authors (Cuzzocrea *et al*., 2005; Kogure *et al*., 2010; Rovensky *et al*., 2002; 2008; 2009b).

In this overview, our results obtained in AA with endogenous (coenzyme Q_10_ and carnosine) and synthetic (stobadine and SMe1) antioxidants as well as two selected compounds from plants (pterostilbene and pinosylvin) will be completed with new unpublished results. The aim is to demonstrate the potential of antioxidant compounds for their use in combination therapy with Mtx.

## Methods

### Animals, experimental design and treatments

Male Lewis rats weighing 160–180 g were obtained from the Breeding Farm Dobra Voda, Slovakia. The rats had free access to standard pelleted diet and tap water. The experimental protocol was approved by the Ethics Committee of the Institute of Experimental Pharmacology and Toxicology and by the Slovak State Veterinary and Food Administration. Adjuvant arthritis (AA) was induced by a single intradermal injection of heat-inactivated *Mycobacterium butyricum* in incomplete Freund's adjuvant (Difco Laboratories, Detroit, MI, USA). The injection was performed near the tail base. The experiments included healthy animals (HC), arthritic animals not treated (AA), arthritic animals treated with methotrexate in the oral dose of 0.3 mg/kg b.w. twice a week (AA-Mtx), arthritic animals treated with coenzyme Q_10_ (CoQ_10_) in the daily oral dose of 20 mg/kg b.w (AA-CoQ_10_), arthritic animals treated with carnosine in the daily oral dose of 150 mg/kg b.w (AA-Carn), arthritic animals treated with the combination of CoQ_10_ and methotrexate (AA-CoQ_10_+Mtx), arthritic animals treated with stobadine dipalmitate (AA-Stb) or its derivative SMe1.2HCl (AA-SMe1) in the oral daily dose of 15 mg/kg b.w., arthritic animals treated with the combination of stobadine dipalmitate and methotrexate (AA-Stb+Mtx), arthritic animals treated with pinosylvin (AA-Pin) or pterostilbene (AA-Pte) in the oral daily dose of 30 mg/kg b.w., arthritic animals treated with the combination of pinosylvin and methotrexate (AA-Pin+Mtx). In the latter combination, arthritic animals were treated twice a week with methotrexate in the oral dose of 0.4 mg/kg b.w. and daily with pinosylvin in the oral dose of 50 mg/kg b.w.. Monotherapy was performed with the same doses.

Methotrexat® Lachema 50 sol. inj. was used. CoQ_10_ in the form of Li-Q-Sorb® was purchased from Tishcon Corp., USA. Carn was purchased from Hamary Chemicals Ltd., Japan. The pyridoindoles were synthetized by Ing. Vladimir Snirc, PhD. Pin and Pte were synthetized in the Institute of Organic Chemistry and Biochemistry, Prague, Czech Republic.

In each experimental group 8–10 animals were used. The duration of the experiments was 28 days. After the animals had been sacrificed under deep ketamin/xylasine anesthesia, blood for plasma preparation and tissue for hind paw joint homogenate preparation were taken on day 28. Plasma was stored at minus 70 °C until biochemical and immunological analysis.

### Clinical parameter evaluated: hind paw volume

We monitored the basic clinical parameter: hind paw volume (HPV). The HPV increase was calculated as the percentage increase in HPV on the experimental day 28 relative to the HPV at the beginning of the experiment. Hind paw volume was recorded with the use of an electronic water plethysmometer (UGO BASILE, Comerio-Varese, Italy).

### Rat IL-1α assay in plasma

For determination of IL-1α in plasma an ELISA kit from Bender MedSystems was used as described in the product manual Rat IL-1α ELISA BMS627 and BMS627TEN. The rat IL-1 ELISA is an enzyme-linked immunosorbent assay for quantitative detection of rat IL-1. Rat IL-1 present in the samples binds to anti-rat IL-1antibodies adsorbed to the microwells. The reaction of a secondary biotin-conjugated anti-rat IL-1 antibody is evaluated by Streptavidin-HRP. Tetramethyl-benzidine oxidation with HRP bound to the immune complex was measured at 490 nm against reference wavelength of 620 nm. The results were calculated from a standard calibration curve obtained for internal standards.

### Tissue activity of cellular γ-glutamyltransferase from hind paw

The activity of cellular γ-glutamyltransferase (GGT) in hind paw joint tissue homogenate was measured by the method of Orlowski & Meister (1970) as modified by Ondrejickova *et al*. (1993). Samples were homogenized in a buffer at 1: 9 w/v (buffer composition: 2.6 mM NaH_2_HPO_4_; 50 mM Na_2_HPO_4_; 15 mM EDTA; 68 mM NaCl; pH 8.1) by Ultra Turax TP 18/10 (Janke & Kunkel, Germany) for 1 min at 0 °C. Substrates (8.7 mM γ-glutamyl-p-nitroanilide (γ-GPN); 44 mM methionine) were added in 65% isopropylalcohol to final concentrations of 2.5 mM and 12.6 mM, respectively. After incubation for 60 min at 37 °C, the reaction was stopped with 2.3 ml cold methanol and the tubes were centrifuged for 20 min at 5000 rpm. Absorbance of supernatant was measured in a Hewlett Packard Vectra 286/12 spectrophotometer in 0.5 cm cuvette at 406 nm. Reaction mixtures in the absence of either the substrate or acceptor were used as reference samples.

### Thiobarbituric acid reactive substances in plasma

TBARS were measured in heparinized blood plasma (Brown & Kelly, 1996). The amount of 750 µl of 0.67% thiobarbituric acid, 750 µl of 20% trichloroacetic acid, 350 µl of phosphate buffer (pH 7.4) were added to 150 µl of plasma, then mixed and incubated in a water bath at 90 °C for 30 min. The reaction was stopped by dipping the test tubes into ice for 10 min. Samples were centrifuged at 3 000 rpm. The supernatant was removed and absorbance measured at 535 nm in a 0.5 cm cuvette.

### Statistical analysis of data

The data for all parameters are expressed as arithmetic mean±S.E.M. For significance calculations unpaired Student‘s *t*-test was used with *p<0.05 (significant), *p<0.01 (very significant), ***p<0.001 (extremely significant). The arthritis group was compared with healthy control animals (*). The treated arthritis groups were compared with untreated arthritis (+). The combination treatment was compared to individual Mtx treatment (#).

## Results

**Figure 1:**
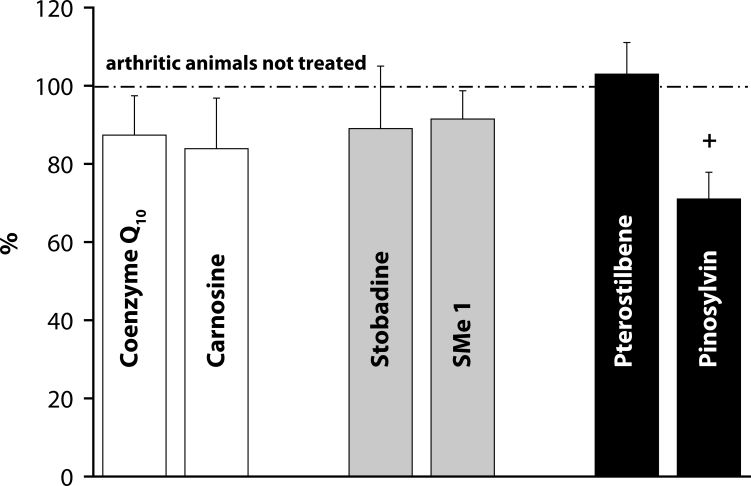
Reduction of hind paw volume on day 28 – treatment with different antioxidants. Values are given as arithmetic mean of percentage ± S.E.M. of reduction of hind paw volume calculated to untreated arthritic rats (100% represented by dot-anddash line). Statistical significance was evaluated using unpaired Student′s t-test: +*p<*0.05 with respect to untreated arthritic animals.

**Figure 2:**
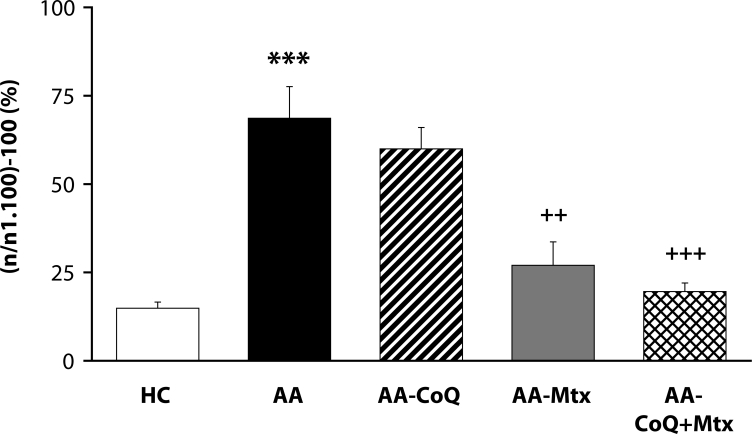
Effect of combined therapy of methotrexate and coenzyme Q_10_ on hind paw volume assessed on day 28. Values are given as arithmetic mean ± S.E.M. Statistical significance was evaluated using unpaired Student′s t-test: ***p<0.001 with respect to control healthy animals, ++*p<*0.01 and +++*p<*0.001 with respect to untreated arthritic animals. The experiment included healthy control animals (HC), arthritic animals without any drug administration (AA), and arthritic animals with the administration of coenzyme Q_10_ (AA-CoQ), methotrexate (AA-Mtx) and combination of methotrexate and coenzyme Q_10_ (AA-CoQ+Mtx).

**Figure 3:**
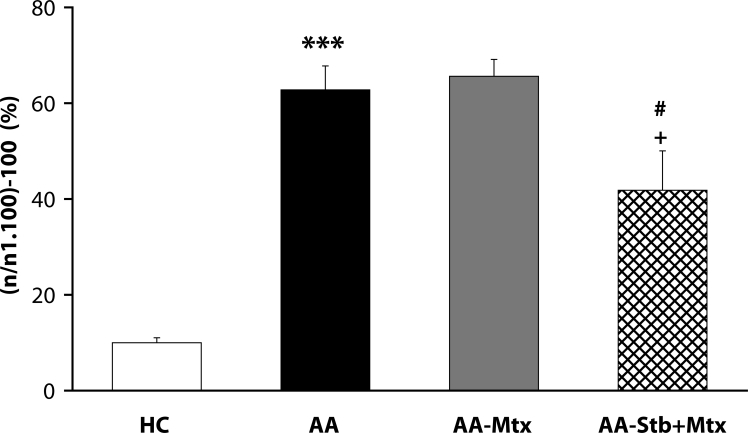
Effect of combined therapy of methotrexate and stobadine on hind paw volume assessed on day 28. Values are given as arithmetic mean ± S.E.M. Statistical significance was evaluated using unpaired Student′s t-test: ***p<0.001 with respect to control healthy animals, +*p<*0.05 with respect to untreated arthritic animals and #*p<*0.05 for comparison of methotrexate monotherapy with combined therapy. The experiment included healthy control animals (HC), arthritic animals without any drug administration (AA), and arthritic animals with the administration of methotrexate (AA-Mtx) and combination of methotrexate and stobadine (AA-Stb+Mtx).

**Figure 4:**
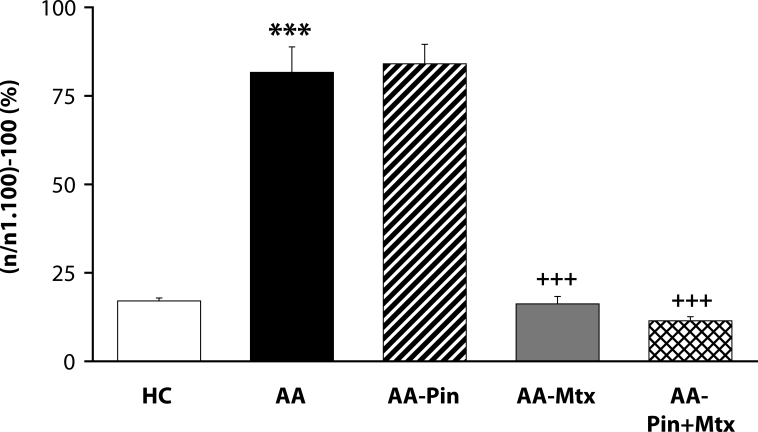
Effect of combined therapy of methotrexate and pinosylvin on hind paw volume assessed on day 28. Values are given as arithmetic mean ± S.E.M. Statistical significance was evaluated using unpaired Student′s t-test: ***p<0.001 with respect to control healthy animals, +++*p<*0.001 with respect to untreated arthritic animals. The experiment included healthy control animals (HC), arthritic animals without any drug administration (AA), and arthritic animals with the administration of pinosylvin (AA-Pin), methotrexate (AA-Mtx) and combination of methotrexate and pinosylvin (AA-Pin+Mtx).

**Figure 5:**
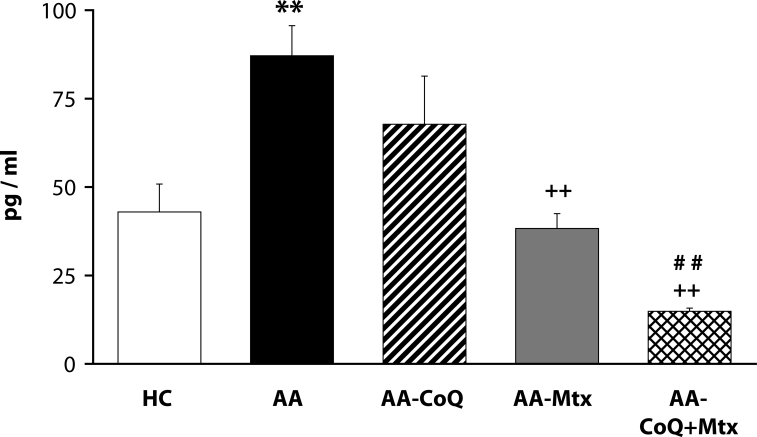
Effect of combined therapy of methotrexate and coenzyme Q_10_ on plasmatic level of IL-1α assessed on day 28. Values are given as arithmetic mean ± S.E.M. Statistical significance was evaluated using unpaired Student′s t-test: **p<0.01 with respect to control healthy animals, ++*p<*0.01 with respect to untreated arthritic animals and ##*p<*0.01 for comparison of methotrexate monotherapy with combined therapy. The experiment included healthy control animals (HC), arthritic animals without any drug administration (AA), and arthritic animals with the administration of coenzyme Q_10_ (AA-CoQ), methotrexate (AA-Mtx) and combination of methotrexate and coenzyme Q_10_ (AA-CoQ+Mtx).

**Figure 6:**
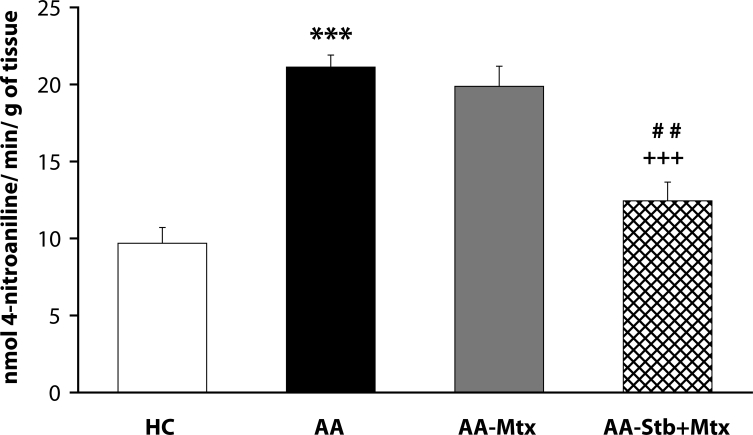
Effect of combined therapy of methotrexate and stobadine on activity of GGT in joint homogenate assessed on day 28. Values are given as arithmetic mean ± S.E.M. Statistical significance was evaluated using unpaired Student′s t-test: ***p<0.001 with respect to control healthy animals, +++*p<*0.001 with respect to untreated arthritic animals and ##*p<*0.01 for comparison of methotrexate monotherapy with combined therapy. The experiment included healthy control animals (HC), arthritic animals without any drug administration (AA), and arthritic animals with the administration of methotrexate (AA-Mtx) and combination of methotrexate and stobadine (AA-Stb+Mtx).

**Figure 7:**
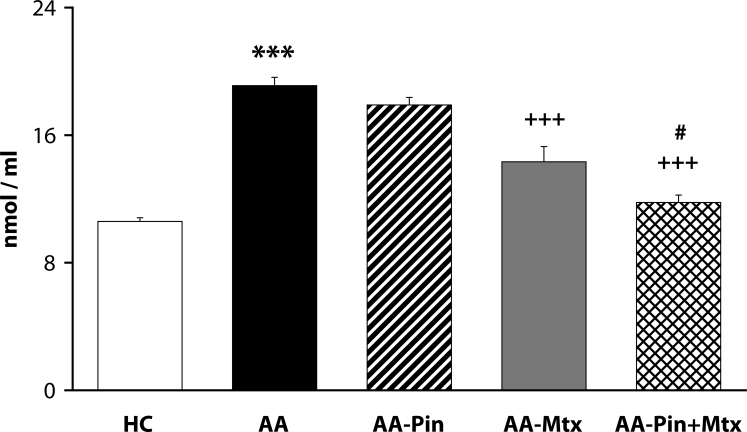
Effect of combined therapy of methotrexate and pinosylvin on plasmatic level of TBARS assessed on day 28. Values are given as arithmetic mean ± S.E.M. Statistical significance was evaluated using unpaired Student′s t-test: ***p<0.001 with respect to control healthy animals, +++*p<*0.001 with respect to untreated arthritic animals and #*p<*0.05 for comparison of methotrexate monotherapy with combined therapy. The experiment included healthy control animals (HC), arthritic animals without any drug administration (AA), and arthritic animals with the administration of pinosylvin (AA-Pin), methotrexate (AA-Mtx) and combination of methotrexate and pinosylvin (AA-Pin+Mtx).

Of the antioxidants administered in monotherapy to arthritic rats only pinosylvin showed a significant reduction of HPV. There were no big differences in the effectivity of antioxidants in the groups compared (CoQ *versus* Carn, and Stb *versus* SMe1), except in the group of stilbenoids where Pin exhibited a notably higher efficacy than Pte (Figure 1). In all combination therapies, HPV was more effectively reduced than by Mtx alone. This effect was most noticeable for the combination of Mtx and Stb (Figures 2–4). All combination therapies achieved a significantly higher efficacy in affecting selected immunological (IL-1α – Figure 5) and biochemical (GGT – Figure 6 and TBARS – Figure 7) parameters compared to Mtx alone.

## Discussion

Rheumatoid arthritis (RA) is a common severe joint disease affecting all age groups. It is thus of great importance to develop new strategies for its treatment. As a number of disease-modifying anti-rheumatic drugs (DMARDs) often have side effects at high doses and/or during long-term administration, increased efficacy without increased toxicity is expected for combination therapy of RA. Methotrexate (Mtx), a folic acid antagonist, has become the predominant immunosuppressive agent used in the treatment of patients with RA (Williams *et al*., 1985). Mtx acts mainly on actively proliferating cells during the S-phase of proliferation, suppresses macrophage function, modulates interleukin-1 (IL-1) and superoxide anion production, and inhibits neutrophil chemotaxis (Moreland *et al*., 1997). Furthermore, Mtx treatment was shown to decrease synovial collagenase gene expression in patients with RA (Genestier *et al*., 2000). The use of Mtx has been limited by some of its toxic manifestations, such as abdominal discomfort, alopecia, oral ulcerations, and cytopenia (Alarcon *et al*., 1989). In clinical studies, infliximab or etanercept have been used in combination with methotrexate to produce greater efficacy of the treatment of RA (Maini *et al*., 1998; Weinblatt *et al*., 1999). TNF-α blockers may be used alternatively with other candidates for RA combination therapy. As resulted from our previous experiments (Bauerova *et al*., 2005a; 2005b; 2008a; 2008b; 2009; Drábikova *et al*., 2009; Dráfi *et al*., 2010; Jančinova *et al*., 2009; Kogan *et al*., 2005; Mačičkova *et al*., 2010; Poništ *et al*., 2010; Sotnikova *et al*., 2009; Štrosova *et al*., 2008; 2009), all performed in the adjuvant arthritis model, substances with antioxidant properties have a high potency to be used in combination therapy with Mtx.

For our experiments, we chose substances with anti-oxidative properties and low toxicity. Administration of CoQ_10_ significantly improved changes of rat body mass, reduced HPV, and prevented CoQ imbalance in skeletal muscle mitochondria. The significantly improved myocardium mitochondrial function indicated that CoQ_10_ supplementation may protect cardiac function also in patients with RA (Bauerova *et al*., 2008b). Based on our results with mitochondrial energetics modification and the observed antiinflammatory and antioxidant effects (Gvozdjakova *et al*., 2004; Bauerova *et al*., 2005a; 2008b; Poništ *et al*., 2007), CoQ_10_ was selected as a candidate for combinatory therapy of RA. Another endogenous antioxidant used – carnosine, an essential endogenous molecule, has many physiological functions: radical scavenging, pH buffering, heavy metal chelating, anti-glycating, and neutralizing toxic aldehydes. Carn was found to have neuroprotective, hepatoprotective, cataract treating, and anti-aging abilities (Boldyrev, 2005), but its antiinflammatory potential in autoimmune systemic inflammatory diseases, as RA, has been scarcely investigated as yet. The first experiment with Carn in AA was performed in our laboratories. Carn beneficially affected HPV measured in time profile (14, 21 and 28 days), significantly on day 14 when the clinical manifestation of the disease started. Carn was able to delay the disease onset, however it was not so effective later on (days 21 and 28) when AA was fully developed. The markers of redox imbalance in plasma (TBARS and protein carbonyls) were significantly decreased, indicating the ability of Carn to restore redox balance *in vivo* (Dráfi *et al*., 2010). Stobadine dipalmitate (Stb) belongs to the group of naturally occurring carbolines (pyridoindoles – PI), tricyclic compounds of indole structure with a side ring comprising another nitrogen atom. Unlike α- and β-carbolines, it does not reveal any obvious toxic effects and possesses antioxidant activity. Stb, (–)-*cis*-2,8-dimethyl-2,3,4,4a,5,9b-hexahydro-1*H*-pyrido[4,3b]indole, was developed and synthesized in a search for new antidysrhythmic drugs at the Institute of Experimental Pharmacology, Slovak Academy of Sciences in Bratislava, Slovakia, in 1983 (Štolc *et al*., 1983). Later also the PI derivative SMe1.2HCl: 8-methoxy-2,3,4,4a,5,9b-hexahydro-1*H*-pyrido[4,3b]indole was synthesized at our institute. The first results with Stb in rat AA were obtained when its protective effect against indomethacin-induced gastroenteropathy was evaluated (Bauerova *et al*., 2004).

Pinosylvin [3′,5′-dihydroxystilbene] and pterostilbene [3,5-dimethoxy-4′-hydroxystilbene] are natural substances from the stilbenoid group, wide-spread in a variety of plants. They are chemically related to resveratrol. Both substances studied inhibited significantly the chemiluminescence (CL) of whole human blood and the CL of isolated human neutrophils (Perečko *et al*. 2008). The new information on the inhibitory effect of Pin and Pte on HPV, ROS and MPO activity suggests that the protective effect of Pin may be beneficial in controlling inflammation in experimental AA (Mačičkova *et al*., 2010).

The situation for adjuvant arthritis is complicated due to the dominant involvement of Th-1-driven autoimmune etiopathology. Oxidative stress (OS) in this animal model occurs as areaction to autoimmune processes. Under these conditions, control of OS is of secondary importance, although it could enhance immunomodulatory therapy of RA. We therefore administered CoQ_10_ (Bauerova *et al*., 2010) or Stb (our unpublished results) or Pin in combination with Mtx (Jančinova *et al*., 2010 and our unpublished results). The basic clinical parameter – HPV – was improved independently of the type of antioxidant used, although Pin seems to be more active than Stb or CoQ_10_ (Figure 1). Interestingly, on comparing all pairs of substances tested, namely CoQ_10_ with Carn, Stb with SMe1, and Pin with Pte (Figure 1), there were no significant differences between the two endogenous antioxidants or two pyridoindoles, but the two stilbenoids exhibited a great difference in their capacity to suppress the hind paw edema in AA. In all combination treatments, HPV was more effectively reduced than by individual Mtx treatment (Figures 2–4).

For further evaluation of the efficacy of CoQ_10_+Mtx administration, we analyzed IL-1α in plasma, which is one of the most important pro-inflammatory cytokines. IL-1 is closely related to inflammation and articular damage in several arthritis models and it is therefore generally accepted that IL-1 has a pivotal role in the pathophysiology of rheumatoid arthritis. In particular, IL-1 is a potent stimulator of synoviocytes, chondrocytes and osteoblasts. Moreover, IL-1 is a key mediator of synovial inflammation and pannus formation (Dinarello & Moldawer, 2002). Figure 5 shows the effects of the given treatments on AA-increased IL-1α levels. The improving effect on the increased cytokine plasmatic levels is rising in the order CoQ_10_, Mtx and CoQ_10_+Mtx. Furthermore, a statistically significant difference favored the combination of Mtx with CoQ_10_, compared to Mtx monotherapy.

GGT is an important component of inflammatory processes since its activity is closely connected with the overall antioxidant status of the organism. We found that the activity of GGT was approx. 3–6 times higher in AA animals than in healthy controls in the spleen and 1.4–2.3 higher in the joint (Bauerova *et al*., 2006; 2008a; 2009; Sotnikova *et al*., 2009). We assume that the increased activity of GGT in AA is a result of elevated systemic oxidative stress. Stb was significantly effective in suppressing the increased activity of GGT in the joint, as expected from its antioxidant potential (Poništ *et al*., 2010). The addition of Stb to Mtx caused significant improvement in lowering this biochemical parameter compared to Mtx monotherapy (Figure 6).

A frequently used marker of lipid peroxidation is MDA assessed as an adduct with TBA. Clinical studies have shown increased plasmatic levels of MDA in patients with RA (Baskol *et al*., 2005; 2006; Sarban *et al*., 2005). In animal models of AA, the level of MDA was elevated in the plasma of arthritic animals (Tastekin *et al*., 2007; He *et al*., 2006; Bauerova *et al*., 2008a; 2009; Štrosova *et al*., 2008; 2009; Sotnikova *et al*., 2009). The combination of Pin with Mtx was more effective in decreasing the plasmatic level of TBARS than was Mtx alone (Fig. 7). This finding is in good agreement with the results of Jančinova *et al*. (2010) showing the combination of the two substances as more active in suppressing spontaneous and stimulated chemiluminescence in blood of arthritic rats than achieved with Mtx administered alone.

Our results obtained with different antioxidants in monotherapies showed reduction of OS in adjuvant arthritis independently of the chemical structure of the compounds or their solubility in lipids (CoQ_10_) or water (Carn). Thus we may conclude that therapeutic benefits from antioxidant treatment are primarily bound to reduction of systemic OS. All combinations tested showed a higher efficacy in affecting biochemical or immunological parameters than Mtx administered in monotherapy. These results indicate that application of supporting immunosuppresive treatment represented by Mtx combined with an antioxidative substance appears to be a good decision. In our future experiments we will try to lower the dose of Mtx in combinations with the most effective antioxidants. We expect that a suboptimal dose of Mtx with an antioxidant could yield an as good efficacy as the optimal dose of Mtx alone in reducing of hind paw edema in AA. An effective combination of an antioxidant with a lowered dose of Mtx could also reduce the toxic side effect of Mtx treatment. Conclusion: We demonstrated the benefit of antioxidant compounds for their use in combination therapy with methotrexate.
